# Extracorporeal shock wave lithotripsy: retrospective study on possible predictors of treatment success and revisiting the role of non-contrast-enhanced computer tomography in kidney and ureteral stone disease

**DOI:** 10.1007/s00240-024-01570-7

**Published:** 2024-04-17

**Authors:** Beatriz Oliveira, Bernardo Teixeira, Martinha Magalhães, Nuno Vinagre, Avelino Fraga, Vítor Cavadas

**Affiliations:** Unidade Local de Saúde de Santo António, Porto, Portugal

**Keywords:** Kidney stones, Ureteral stones, Shock wave lithotripsy, Treatment outcome

## Abstract

Extracorporeal shock wave lithotripsy (ESWL) is a safe and efficient treatment option for urinary stone disease. The overall stone-free rate (SFR) varies significantly. This study aimed to assess the influence of stone size, location, stone density, and skin-to-stone distance (SSD), on the outcome of ESWL. We assessed whether pre-treatment non-contrast-enhanced CT scan (NCCT) confers significant advantages compared to kidney-ureter-bladder film (KUB) only. We reviewed the medical records of 307 cases (165 men, 142 women) with renal and ureteral stones treated consecutively at our institution with ESWL between 2020 and 2023. 44 of these underwent a NCCT. The outcome of ESWL was defined in two ways: visible stone fragmentation on KUB, and the need for further treatment. Overall success of fragmentation was 85% (261 patients). 61% of patients (*n* = 184) didn’t need any further treatment. Stone size and location correlated significantly with treatment outcomes regarding the need for further treatment (*p* = 0.004) and stone fragmentation (*p* = 0.016), respectively. Unlike mean SSD (*p* = 0.462), the mean attenuation value (MAV) significantly correlated with the need for retreatment (*p* = 0.016). MAV seems to be a better predictor of treatment success (AUC of the ROC curve: 0.729), compared to stone size (AUC: 0.613). The difference between groups (with and without NCCT) in both treatment outcomes did not reach statistical significance. During decision-making, information regarding SSD and MAV can be useful in more dubious scenarios. However, it appears that their inclusion doesn’t provide substantial advantages when compared to relying solely on KUB.

## Introduction

Extracorporeal shock wave lithotripsy (ESWL) stands as a non-invasive, safe, and effective treatment choice for renal and ureteral stones. The success of ESWL depends on the procedure itself (namely the efficacy of the lithotripter and performance of ESWL), patients’ habitus, and stone characteristics (size, location, and composition). ESWL can reach Stone Free Rates (SFRs) of around 80% [1]. A recent meta-analysis reported ESWL as being less effective than percutaneous nephrolithotomy (PNL) and retrograde intrarenal surgery (RIRS) [2]; for urinary stones < 1 cm, there is no discernible difference in SFRs between ESWL and flexible ureteroscopy [1]. The higher rate of secondary procedures is the main obstacle for ESWL, compared to RIRS or PNL [3]. If patients are not selected adequately, SFRs can decrease, making retreatment potentially necessary. This consequently increases medical costs and unnecessary exposure to shock waves and radiation. What may fail in the selection of patients for ESWL is an incomplete stone evaluation. When patients are proposed for ESWL directly through their emergency room visit in our institution, most of them are evaluated through ultrasound and kidneys, ureters, and bladder film (KUB). This usually allows the patient to be quickly referred for an ESWL treatment. Stone disease is therefore not completely characterized regarding stone composition and inner structure, and also the patient’s habitus regarding skin-to-stone distance and surrounding anatomy. Still, it is possible through KUB to measure the largest stone size and to identify stone location, which are characteristics that also have a predictive value of treatment failure.

In the past decade, most studies have focused on assessing the impact of characteristics evaluated through non-contrast-enhanced CT scans (NCCT) on stone fragmentation. To date, eight studies have concurrently assessed renal and ureteral stone disease using NCCT [4–11]. Factors such as stone size, stone density, and skin-to-stone distance (SSD) are recognized as significant correlates of treatment success. However, there remains a lack of standardization in defining treatment outcomes across most studies. While some define treatment success as achieving a stone-free status [8, 11–15] others define it as visible stone fragmentation on KUB [16], with follow-up periods ranging from two weeks to three months. Moreover, there is variability in the definition of a complete ESWL treatment, with some authors considering up to three ESWL treatments as treatment success, while others define success with just one treatment. Table 1 presents a review of the literature.

We aim to investigate the possible predicting factors of ESWL success obtained with two different imaging modalities (KUB or NCCT). We correlate these variables with different treatment outcomes, namely stone fragmentation on KUB after treatment, and the need for retreatment after one session of ESWL.

**Table 1:** Review of the literature. A: stone-free status; B: fragments ≤ 3 mm; C: fragments ≤ 4 mm; D: fragments ≤ 5 mm; E: visible fragmentation on KUB

Authors	Year	Stone location	n All/ Renal/ Ureteral	Treatment success	Sessions	Success rate	Prediction of treatment outcome (All/Renal/Ureteral)					
							Stone size	Stone location	BMI	SSD	MAV	Cut-offs
Pace KT et al. [12]	2000	Ureteral	1588/-/1588	A	> 1	68%	Yes/-/Yes	Yes/-/Yes	-/-/-	./-/-	-/-/-	
Joseph P et al. [17]	2002	Renal	30/30/-	B	> 1	80%	No/No/-	-/-/-	No/No/-	-/-/-	Yes/Yes/-	MAV: 950 HU
Pareek G et al. [4]	2003	Renal and ureteral	50/20/30	B	Only 1	64%	-/-/.	-/-/.	No/No/-	-/-/-	Yes/Yes/Yes	MAV (ureteral): 900 HU
Wang LJ et al. [18]	2005	Renal	80/80/-	C	> 1	52.5%	Yes/Yes/-	-/-/.	-/-/-	-/-/-	Yes/Yes/-	MAV (renal): 900 HU
Gupta NP et al. [5]	2005	Renal and ureteral	108/89/19	D	> 1	76%	Yes/-/-	-/-/-	-/-/-	-/-/-	Yes/-/-	MAV: 750 HU
Yoshida S et al. [6]	2006	Renal and ureteral	56/25/31	B	> 1	70%	Yes/Yes/-	No/No/-	-/-/-	-/-/-	Yes/-/-	-
Chi-Fai Ng et al. [13]	2007	Renal and ureteral	2489/1498/694	A	Only 1		Yes/-/-	Yes/-/-	-/-/-	-/-/-	-/-/-	-
El-Nahas AR et al. [19]	2007	Renal	120/120/-	C	> 1	87.5%	-/-/-	No/No/-	Yes/Yes/-	Yes/Yes/-	Yes/Yes/-	MAV: 1000 HU
Perks AE et al. [20]	2008	Renal	111/111/-	D	Only 1	64%	Yes/Yes/-	No/No/-	No/No/-	Yes/Yes/-	Yes/Yes/-	MAV: 900 HU; SSD: 9 cm
Ng CF et al. [21]	2009	Ureteral	94/-/94	A	Only 1	50%	-/-/-	-/-/-	No/-/No	Yes/-/Yes	Yes/-/Yes	MAV: 593 HU; SSD: 9.2 cm
Patel T et al. [14]	2009	Renal	83/83/-	A	Only 1	61.4%	No/No/-	No/No/-	-/-/-	Yes/Yes/-	No/No-	SSD: 10 cm
Wiesenthal JD et al. [7]	2010	Renal and ureteral	422/218/204	C	Only 1	62.8%	-/-/-	No/No/No	Yes/No/Yes	Yes/Yes/Yes	Yes/Yes/Yes	MAV: 900 HU; SSD: 11 cm
Park YI et al. [22]	2010	Renal	115/115/-	B	> 1	68.7%	Yes/Yes/-	No/No/-	-/-/-	No/No/-	Yes/Yes/-	MAV: 863 HU
Shah K et al. [8]	2010	Renal and ureteral	99/71/28	A	Only 1	-	-/-/-	No/No/No	-/-/-	-/-/-	Yes/-/-	-
Choi JW et al. [23]	2012	Ureteral	153/-/153	C	> 1	83%	Yes/-/Yes	No/-/No	No/-/No	-/-/-	No/-/No	-
Tanaka M et al. [9]	2013	Renal and ureteral	75/27/48	C	> 1	73.3%	-/-/-	No/-/-	No/-/-	No/-/-	Yes/-/-	MAV: 780 HU
Nakasato T et al. [10]	2015	Renal and ureteral	260/92/168	C	Only 1	66.5%	Yes/Yes/Yes	Yes/Yes/Yes	-/-/-	No/-/-	Yes/-/-	MAV: 815 HU
Müllhaupt G et al. [16]	2015	Ureteral	104/-/104	E	> 1	50%	No/-/No	No/-/No	Yes/-/Yes	Yes/-/Yes	No/-/No	SSD: 119 mm; BMI: 25.9 Kg/m2; Weight: 82.5 Kg
Celik S et al. [11]	2015	Renal and ureteral	254/123/131	A	> 1	-	Yes/Yes/Yes	-/-/-	-/Yes/-	-/Yes/-	Yes/Yes/Yes	-
Wu H et al. [24]	2016	Renal	376/376/-	C	> 1	-	-/-/-	Yes/Yes/-	No/No/-	-/-/-	-/-/-	-
Kang DH et al. [25]	2016	Ureteral	680/-/680	A	Only 1	63.1%	Yes/-/Yes	No/-/No	-/-/-	No/-/No	Yes/-/Yes	Stone size: 10 mm / MAV: 784 HU
Waqas M et al. [26]	2018	Renal	203/-/203	C	> 1	60.1%	Yes/-/Yes	Yes/-/Yes	No/-/No	Yes/-/Yes	Yes/-/Yes	Stone size: 5–10 mm; SSD: 100 mm.
Yoon JH et al. [27]	2021	Ureteral	150/-/150	C	> 1		Yes/-/Yes	No/-/No	No/-/No	No/-/No	Yes/-/Yes	-
Alić J et al. [28]	2022	Ureteral	115/-/115	C	> 1	82.6%	Yes/-/Yes	No/-/No	Yes/-/Yes	-/-/-	-/-/-	Stone size: 10 mm; BMI: range 18.5–29.9 kg/m²
Elawady H et al. [15]	2022	Renal	100/100/-	A	> 1	70%	-/-/-	-/-/-	No/No/-	Yes/Yes/-	Yes/Yes/-	SSD: 86 mm / MAV: 975 HU

## Methods

We evaluated in this retrospective study 307 consecutive ESWL treatments of renal and ureteral stone disease, between January 2020 and April 2023. We included patients with ureteral and kidney stones and excluded the ones who had more than one stone to treat. Data analysis was made after authorization from the Ethics Committee of Unidade Local de Saúde de Santo António (130-DEFI/122-CE). Stone size was measured by obtaining the maximum length of the stone on KUB. NCCT images were analyzed in a standard bone window (window width-1.120 and window level-300) [16]. We obtained the mean attenuation value (MAV), which defines stone density, by measuring the mean HU of the region of the stone excluding adjacent soft tissue. SSD was measured as defined by Nahas et al. [19], including the value of SSD at 0º, 45º and 90º. The mean SSD was calculated as the average value of those three measurements.

All lithotripsy treatments were performed using Siemens’ MODULARIS Variostar®. In the course of ESWL, patients received analgesia with paracetamol and remifentanil, and were subjected to a maximum of 3,000 shocks, with a frequency of 60 shocks/minute for obese patients and 90 shocks/minute for the remaining patients. The intensity varied according to the location of the stone. The power was incrementally increased during the procedure to allow renal vasoconstriction and patient comfort. In patients with various comorbidities, a lower intensity level was used, along with control of arterial pressure along the treatment. Stones were targeted through bi-planar fluoroscopy at regular intervals. Patients were discharged on the same day after the procedure.

Patients were followed up on an outpatient basis, with a KUB and urology consultation within one month after treatment. In this consult, determination of SFRs, and review of symptoms and complications were made by the attending physician. If necessary, a new treatment was proposed, namely a new session of ESWL, RIRS, or PNL.

We extracted from patients’ records characteristics to be correlated with treatment outcome, namely age, sex, weight, body mass index (BMI), presence or not of a JJ stent, stone size, and location. In patients with NCCT, MAV and SSD at 0º, 45º, and 90º were also included. We defined two variables as treatment outcomes: stone disintegration on KUB at one-month follow-up and need for retreatment.

Univariate (chi-square, t-test, and Mann-Whitney test when appropriate) and multivariate (binary logistic regression) analyses were performed to define factors significantly correlated with treatment outcomes. All tests were two-sided and we defined the p-value as < 0.05 to reject the null hypothesis. Multivariate analysis was performed including variables with marginal association with treatment outcome (*p* < 0.20). Receiver operating characteristic (ROC) curves were generated for factors considered to significantly predict ESWL outcome based on multivariate analysis. Statistical analyses were performed using IBM SPSS Statistics Version 29.0.1.0 (IBM Corp., New York, U.S.A.).

## Results

A total of 307 ESWL treatments were included (165 men, 142 women), being 254 patients in total. Median age was 52 years (range 19–83 years) and BMI was 26.1 kg/m² (range 16.7–28.8 kg/m²). Median stone size was 9 mm (range 3–24 mm). From the analyzed cases, 44 patients underwent a NCCT because they presented with obstructive pyelonephritis in the emergency department (*n* = 23), had previous follow-up with NCCT in urology consultation (*n* = 9), had previous evaluation from an external or family physician (*n* = 10), or there were doubts in the evaluation of X-Ray images (because of bone calcifications) (*n* = 2). In this cohort, the median SSD was 114 mm (range 68–173 mm) and MAV 748 HU (median 270–1185 HU). 56 (18%) of the cases were caliceal stones, 102 (33%) were in the renal pelvis, and 149 (49%) were ureteral. Because only a small number of patients were able to provide the calculus for analysis (*n* = 35), we opted not to include information on stone composition. Regarding SFRs, 126 (41%) cases had no residual fragments (RFs). Nine and 14 cases had RFs of < 2 mm (3%) and between 2 and 4 mm (6%), respectively. 158 cases had RFs of > 4 mm (51%). The rate of complications was 21,5%, as described in Table 2.

**Table 2:** Reported complications of ESWL

Complications	n	%
None	241	78.5%
Renal colic	33	10.7%
Regrowth of residual fragments	4	1.3%
Infections	9	2.9%
Bacteriuria	2	0.7%
Symptomatic haematoma	4	1.3%
Steinstrasse	8	2.6%
Obstructive pyelonephritis -	5	1.6%
Hematuria with urinary retention	1	0.3%

In terms of treatment outcome, stone fragmentation was observed in 261 treatments (85%). Among this subset, 77 (30%) still required additional interventions, ESWL in 60 cases (77%), RIRS in 14 cases (18%), and PNL in three cases (4%). The retreatment rate was 37% (*n* = 115), with 73 undergoing another session of ESWL (23%). Moreover, 13% of cases needed a different treatment modality besides ESWL, with 39 undergoing RIRS (12%) and three undergoing PNL (1%). To summarize, out of the 254 patients enrolled in this study, 212 (83.5%) successfully managed their urinary stone disease solely with ESWL.

Regarding stone fragmentation, only stone location showed a significant correlation to stone fragmentation (*p* = 0.016). In the NCCT cohort, stone density (MAV) tended to be higher in cases with unsuccessful stone fragmentation, without reaching statistical significance (*p* = 0.064). There was no correlation between having done a CT scan before treatment and this treatment outcome (*p* = 0.531). Stone size correlated significantly with the need for retreatment (*p* = 0.004). In the NCCT cohort, unlike mean SSD (*p* = 0.462), MAV showed a significant correlation with no need for retreatment (*p* = 0.016). There was no correlation between having done a CT scan and this treatment outcome (*p* = 0.236). The results of the univariate analysis are summarized in Table 3.

**Table 3:** Results of univariate analysis. Variables with marginal association with treatment outcome were included in the multivariate analysis, the results of which are summarized in Table 4. The ROC curves for stone size and MAV are illustrated in Figs. 1 and 2

Characteristic (all patients)	Successful disintegration	Unsuccessful disintegration	p-value	No need for retreatment	Need for retreatment	p-value
Number of patients (%)	261 (85%)	46 (15%)	-	192 (63%)	115 (37%)	-
Age, years (median, range)	52 (19–82)	55 (31–83)	0.117	51 (19–81)	53 (20–83)	0.352
Gender, M/F (N/%)	139 (45.3%) / 121 (39.4%)	26 (8.5%) / 20 (6.5%)	0.519	101 (32.9%) / 91 (29.6%)	54 (17.6%) / 61 (19.9%)	0.604
Weight, kg (median, range)	73 (43–190)	77 (53–128)	0.196	72 (43–190)	74 (47–128)	0.219
BMI, kg/m2 (median, range)	25.9 (16.7–28.8)	27.1 (20.2–49)	0.134	25.9 (16.7–28.8)	26.9 (20.1–49)	0.061
Stone size, mm (median, range)	9.5 (3.1–24)	9 (3–21)	0.469	9 (3.1–24)	10.2 (3–22)	**0.004**
Location (N, %)			**0.016**			0.224
- renal [upper, median pole]	24 (9.6%) [5 (2%), 19 (7%)]	7 (152%) [4 (8.7%), 3 (6.5%)]	-	21 (10.9%) [3 (1.6%), 18 (9.4%)]	10 (8.7%) [6 (5.2%), 4 (3.5%)]	-
- renal (lower pole)	19 (7%)	5 (10.9%)	-	15 (7.8%)	10 (8.7%)	-
- renal pelvis	95 (33%)	7 (15.2%)	-	60 (31.2%)	42 (36.5%)	-
- ureter [upper/ middle/ lower]	122 (46.7%) [39 (14.9%), 42 (16.1%), 41 (15.7%)]	27 (58.7%) [6 (13%), 8 (17.4%), 13 (28.3%)]	-	96 (50%) [29 (15.1%), 34 (17.7%), 33 (17.2%)]	53 (46.1%) [16 (13.9%), 16 (13.9%), 21 (18.3%)	-
Ureteral stent in place (N, %)	49 (16%)	9 (2.9%)	0.899	36 (11.7%)		0.934
Retreatment (N, %)	77 (30%)	38 (82%)		-	115 (100%)	-
- ESWL	60 (77%)	13 (28.3%)		-	73 (63.5%)	-
- URS	14 (18%)	25 (54.3%)		-	39 (33.9%)	
- PERC	3 (4%)	0		-	3 (2.6%)	-
CT scan for treatment decision, with NCCT / no NCCT (N,%)	36 (14%), 225 (73%)	8 (17.4%), 38 (82.6%)	0.531	24 (12.5%), 168 (87.5%)	20 (17.4%), 95 (82.6%)	0.236
**NCCT cohort** (*n* = 44)						
Number of patients (%)	36 (81%)	8 (19%)	-	24 (55%)	20 (45%)	
Skin-to-stone distance, mm, mean (median, range)	115 (76–168)	128 (71–160)	0.459	115 (76–168)	123 (71–160)	0.462
Skin-to-stone distance, mm, 0° (median, range)	116 (77–190)	130 (87–150)	0.824	116 (77–190)	119 (86–152)	0.535
Skin-to-stone distance, mm, 45° (median, range)	106 (10–165)	116 (91–151)	0.448	107 (10–165)	108 (83–151)	0.711
Skin-to-stone distance, mm, 90° (median, range)	113 (68–173)	128 (83–147)	0.318	114 (68–172)	116 (83–148)	0.34
Mean attenuation value, HU (median, range)	721 (270–1123)	899 (525–1185)	0.064	678 (304–1051)	859 (270–1185)	**0.016**

**Table 4:** Results of multivariate analysis. Exp(B): exponential regression coefficient/ odds-ratio; S.E.: standard error; 95% C.I.: 95% confidence interval; AUC: area under the curve

Variables	Exp (B)	S.E.	95% C.I. for Exp(B)	p-value	ROC curve analysis			
					Sensitivity	Specificity	AUC	Cut-off value
**Stone disintegration** (Hosmer-Lemeshow test: *p* = 0.776) reference category: upper pole								
Stone location: renal pelvis	11.712	0.812	2.386–57.576	0.002	-	-	-	-
Stone location: upper ureter	5.448	0.831	1.069–27.775	0.041	-	-	-	-
**Need for retreatment** (Hosmer-Lemeshow test: *p* = 0.123)								
Largest stone size	1.100	0.034	1.030–1.175	0.005	56.5%	43.8%	0.613	9.75 mm
**Need for retreatment, NCCT cohort** (Hosmer-Lemeshow test: *p* = 0.224)								
MAV	1.005	0.002	1.001–1.008	0.013	60%	83%	0.729	827 HU

**Fig. 1:**
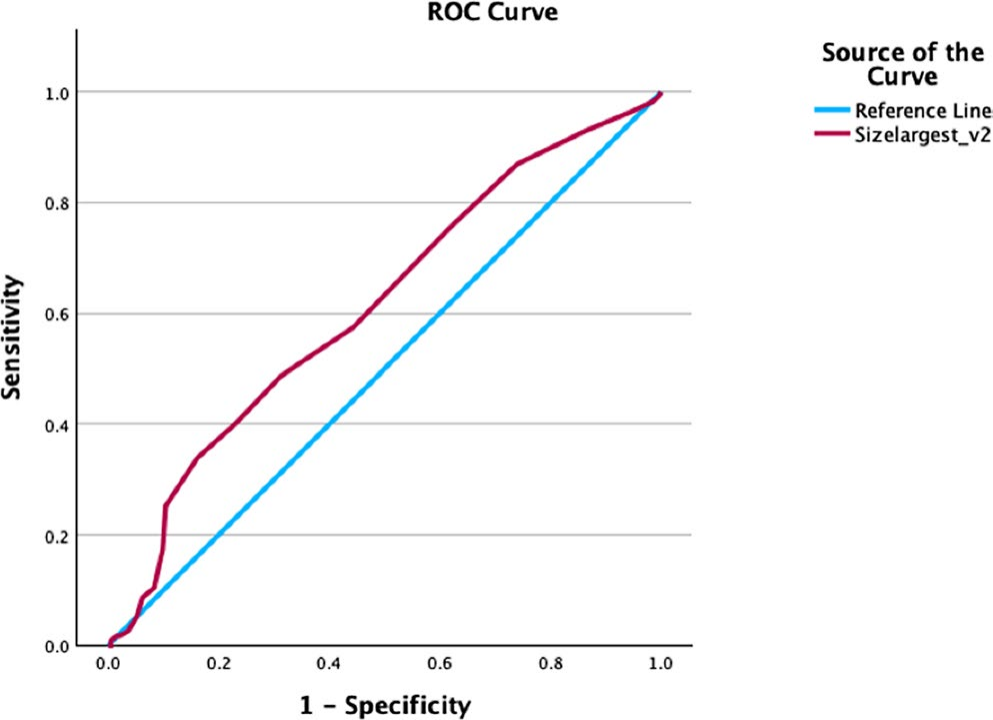
ROC curve for largest stone size

**Fig. 2:**
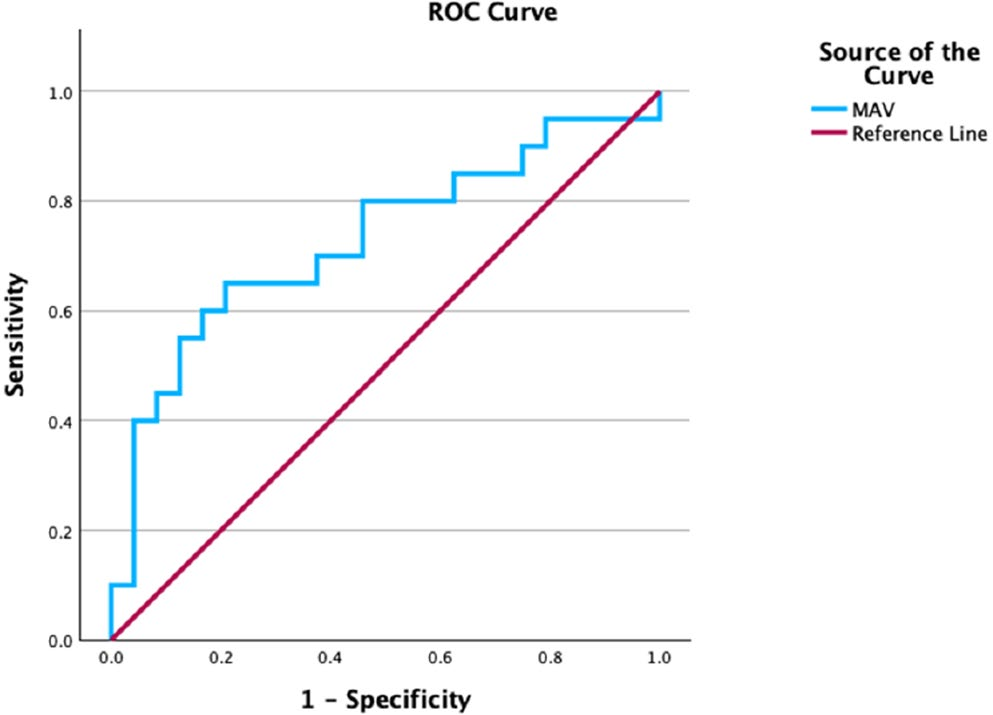
ROC curve for MAV

## Discussion

This is the first study to correlate stone characteristics with two different treatment outcomes. The absence of standardized criteria defining a successful ESWL treatment might account for the varying data observed in prior studies. Our findings notably diverge when comparing the two treatment outcome categories. The only significant variable in our study that could predict stone disintegration was stone location. Nakasato et al., in a retrospective study of 260 patients with renal and ureteral stones, also encountered this correlation, with a better outcome in stones located in the ureter than renal stones [10]. Nevertheless, this latest study defined treatment success as an SFR of < 4 mm at twelve weeks, not solely stone fragmentation. Most published research does not emphasize stone location as a major predictor of stone disintegration; it appears to be more relevant when related to the stone clearance rate. Stones located in the lower renal pole, when treated, tend to leave fragments that remain in the calyx and cause recurrent stone formation. The reported SFR for lower pole calculi is 25–95% [29], compared to the SFR of 32% in our study.

The second outcome defined in our work, the retreatment rate, is probably even more relevant for treatment selection. Stone fragmentation is an important factor when evaluating the effectiveness of ESWL but does not translate to its overall success. This is because even in cases where fragmentation is successful, additional interventions may be necessary if residual fragments exceed 4 mm and the patient continues to experience symptoms. Upon analyzing our findings, 30% (77 cases) of the cohort with successful fragmentation still needed further intervention. The definition of retreatment is, however, different between studies. The overall success of ESWL in our study increases from 63 to 83.5%, if we set the retreatment outcome including or excluding another session of ESWL, respectively. There is a trend towards a higher success rate in studies that consider treatment to be successful when they include one to three ESWL sessions (Table 1).

Our findings indicate that the maximum stone size measured on KUB can predict the need or retreatment, aligning with the majority of literature. It’s worth noting that we measured size on KUB rather than NCCT, which may lead to discrepancies in results due to its lower sensitivity. However, other studies have also measured stone size in KUB and had similar results. One such example is the study by Ng et al. in which 2489 patients were assessed using ultrasound and KUB alone. A statistically significant correlation (*p* < 0.001) was also found between size and treatment success (stone-free status after one session at three months of follow-up) [13]. The study by Perks et al. measured stone size with KUB and NCCT, with similar results in terms of correlation to stone fragmentation [20].

NCCT is a widely accepted imaging method to characterize stone disease; the information obtained through this method, namely stone density, size, and SSD, has been widely studied and related to ESWL efficacy. The preliminary studies of Joseph et al. [17] and Pareek et al. [4] first described a significant correlation between stone disintegration and MAV. After these, a relevant part of the discussion in the literature was related to the standardization of MAV measurement [6]. We adopted the same method as Müllhaupt et al., defining regions of interest just smaller than the stone in magnified images [16]. Different measuring methods can justify distinct results in the literature. As in the study by Müllhaupt et al., our study did not show any correlation between MAV and stone disintegration. However, when the defined treatment outcome was the need for retreatment, a significant correlation was found, with a cut-off value of 827 HU. This result is similar to the one described by Wiesenthal et al., in which the treatment outcome was also defined as a single-treatment success, with a cut-off value of 900 MAV [7].

The measuring method of SSD did not vary among studies [19]. In our results, SSD could not predict treatment outcomes in both its definitions. Although the majority of literature supports the predictive value of SSD in treatment success, some studies align with our findings. Yl et al. conducted a retrospective study of 115 patients with renal stones who underwent more than one ESWL session. They found no correlation between SSD and an SFR of < 3 mm at one-month follow-up (*p* = 0.501) [22]. Another example is the study by Kang et al., also with a retrospective nature, that evaluated 399 patients with ureteral stones who underwent a single ESWL session [25].

An important consideration is whether NCCT is strictly necessary for treatment decision. It is generally accepted that NCCT allows for a superior assessment of stone disease. However, good patient selection also seems to be possible using KUB alone, namely assessing stone size. In our study, the sensitivity and specificity of MAV as a predictor of treatment success is higher compared to stone size alone; the AUC of the ROC curve is 0.729, compared to the AUC of stone size, 0.613. However, the difference between groups in both treatment outcomes did not reach statistical significance. Also, the predicting value of stone size demonstrated in our study seems to be lower compared to the results of other studies. In the prospective study by Wang et al. that included 80 patients with renal stones, both stone size and stone density were significant predictors of treatment outcome (SFR ≤ 4 mm in NCCT at three months). The AUC for stone size was higher than stone density (0.855 and 0.768, respectively) [18]. Another example is the retrospective study by Park et al., which included 115 patients with renal stones. Here, the AUC between stone density and size did not differ significantly (stone density: 0.874; stone size: 0.827, *p* = 0.388) [22]. On the other hand, in the prospective study from Yoshida et al., which included 62 patients with renal and proximal ureteral stones, MAV had a better negative predictive value than maximal diameter (78.6% compared to 66.7%), being treatment failure residual fragments bigger than 3 mm after three ESWL sessions [6]. Tanaka et al. analyzed 75 patients retrospectively and described MAV as the only independent predictor of ESWL success (AUC of 0.692) [9]. These conflicting results leave room for discussion about whether it is mandatory to assess patients with NCCT for treatment selection.

Our study is not without limitations. Its retrospective nature implies a lack of standardization in assessing patients before treatment, particularly in terms of imaging. This comparison was, nevertheless, one of the aims of this study. NCCT images were acquired using different devices, with collimation adapted to each patient, differing from some prospective studies. Patients were assessed by different physicians using KUB images, usually less sensitive for detecting residual fragments. However, this reflects the usual practice among urologists; we believe that studies should be tailored to the reality of clinical practice.

## Conclusions

The selection of treatment for renal and ureteral stones should prioritize stone location and size, as widely accepted in the scientific community. The choice of imaging modalities for patient evaluation seems to have advantages both ways. In linear cases, KUB offers a fast, easily accessible imaging modality for treatment decision, with a proven value in the prediction of treatment outcome. NCCT provides more precise information for clinicians and patients, which can be useful in cases at high risk of disintegration failure, minimizing unnecessary exposure to shock waves and radiation.

## Data Availability

Data is provided within the manuscript.
